# A case of serotonin syndrome following the reintroduction of aripiprazole after dextromethorphan overdose

**DOI:** 10.1002/pcn5.70162

**Published:** 2025-07-15

**Authors:** Naoko Saito, Nobuhiko Noguchi, Maki Toida, Masatoshi Miyauchi, Takeshi Asami

**Affiliations:** ^1^ Department of Psychiatry Yokohama City University School of Medicine Yokohama Japan; ^2^ Numazu Chuo Hospital Shizuoka Japan

**Keywords:** aripiprazole, dextromethorphan, drug interaction, overdose, serotonin syndrome

## Abstract

**Background:**

Serotonin syndrome is a potentially life‐threatening condition that arises from excessive serotonergic activity. Dextromethorphan (DXM), commonly used as a non‐prescription antitussive, can induce serotonin syndrome when taken in high doses or in combination with other serotonergic agents. At supratherapeutic levels, DXM acts as a serotonin reuptake inhibitor and *N*‐methyl‐D‐aspartate (NMDA) receptor antagonist, thereby significantly increasing the risk of serotonin toxicity.

**Case Presentation:**

We present the case of an 18‐year‐old female diagnosed with attention‐deficit/hyperactivity disorder (ADHD) and comorbid depressive symptoms who developed serotonin syndrome after the reinitiation of aripiprazole in the context of recent DXM overdose. Following ingestion of an estimated 600 mg of DXM, the patient exhibited signs of toxicity. The subsequent administration of aripiprazole was temporally associated with the emergence of serotonin syndrome, evidenced by hyperreflexia, hypertonia, and fever, in accordance with the Hunter Serotonin Toxicity Criteria.

**Conclusion:**

This case illustrates the necessity of cautious reintroduction of serotonergic or dopaminergic medications following DXM overdose. Given the potential for prolonged metabolism and interaction—especially in individuals with reduced CYP2D6 activity—clinicians should allow for adequate washout periods before restarting psychotropic agents, such as aripiprazole.

## BACKGROUND

Serotonin syndrome is a potentially life‐threatening condition that arises from excessive serotonergic activity. Dextromethorphan (DXM), commonly used as a non‐prescription antitussive, can induce serotonin syndrome when taken in high doses or in combination with other serotonergic agents. At supratherapeutic levels, DXM acts as a serotonin reuptake inhibitor and *N*‐methyl‐D‐aspartate (NMDA) receptor antagonist, thereby significantly increasing the risk of serotonin toxicity.

At high doses, DXM's NMDA receptor antagonist properties lead to dissociative and euphoric effects.^1^ DXM is primarily metabolized via cytochrome P450 2D6 (CYP2D6), and its serotonergic properties raise concerns about interactions with other psychiatric medications.^2^ At high doses, DXM can cause serotonin syndrome, particularly when combined with other serotonergic agents.

Serotonin syndrome is a serious condition characterized by autonomic instability, neuromuscular hyperactivity, and altered mental status.^3^ While previously thought to occur primarily with monoamine oxidase inhibitors (MAOIs),^4^ recent reports indicate that DXM alone can induce serotonin syndrome.^5^ Several case reports have documented serotonin syndrome in individuals who overdosed on DXM, especially when combined with CYP2D6 inhibitors, such as fluoxetine or paroxetine. The delayed clearance of DXM due to CYP2D6 inhibition is a key factor that increases the risk of serotonin toxicity in overdose cases. The severity of the syndrome was retrospectively assessed with reference to the Serotonin Syndrome Scale,^6^ although formal scoring was not conducted at the time due to the acute clinical situation.

There have been multiple case reports to date of serotonin syndrome caused by an overdose of DXM, and there have also been reports of cases of serotonin syndrome developing after taking an overdose of the over‐the‐counter cough medicine “Shin‐Kontak Cough Medicine Double Persistence,” which is generally available in Japan.^7^

In Japan, one of the drugs covered by health insurance for depressive symptoms is aripiprazole. Aripiprazole, an atypical antipsychotic, acts as a partial agonist at serotonin 5‐hydroxytryptamine 1 A receptor (5‐HT1A receptor) and as an antagonist at 5‐HT2A receptors. Although it is not commonly associated with serotonin syndrome, its metabolism via CYP2D6 and CYP3A4 suggests a possible interaction with DXM, particularly in poor metabolizers of CYP2D6. In the present case, the effects of aripiprazole on serotonin receptors, combined with the prolonged presence of DXM metabolites, may have contributed to serotonin toxicity.

## CASE PRESENTATION

We present the case of an 18‐year‐old female diagnosed with attention‐deficit/hyperactivity disorder (ADHD) and comorbid depressive symptoms who developed serotonin syndrome after the reinitiation of aripiprazole in the context of recent DXM overdose. Following ingestion of an estimated 600 mg of DXM, the patient exhibited signs of toxicity. The subsequent administration of aripiprazole was temporally associated with the emergence of serotonin syndrome, evidenced by hyperreflexia, hypertonia, and fever, in accordance with the Hunter Serotonin Toxicity Criteria.

The patient was a high school student who had been diagnosed with ADHD during childhood, as she had been hyperactive and inattentive. She was treated with atomoxetine 25 mg/day, but she had depressive symptoms and self‐injurious behavior (self‐cutting), so she was treated with fluvoxamine 50 mg/day, initiated approximately 3 months prior to admission. Due to the closure of schools during the spread of COVID‐19, she became confined to her home. After the epidemic subsided, she started going to school, but as soon as she developed abdominal pain and palpitations, it became difficult for her to go to school. Her depressive symptoms worsened, and the frequency of her self‐injurious behavior increased. She transferred to a correspondence high school, but was unable to attend even the required in‐person classes and instead remained at home. Her lifestyle became disordered and she began to have insomnia. At home, she spent her time playing online games and gradually made more friends online. Later, she began to engage in behaviors such as forming a partner relationship with someone she met online and increasing the frequency of her self‐cutting. Having learned from an acquaintance that excessive intake of DXM may induce dissociative and euphoric effects, she deliberately ingested an estimated 600 mg at one time in an attempt to alleviate psychological distress. This dose is well above the therapeutic range and known to produce psychotropic effects, increasing the risk of serotonin toxicity.

After an emergency admission, she presented with signs of DXM toxicity, including dissociation, ataxia, and mild autonomic instability. Psychiatric evaluation was performed, and she was voluntarily admitted to the psychiatric department for further evaluation and treatment. Given her impulsivity and history of substance misuse, it was thought that psychological education regarding the safety of drugs was essential. She also exhibited heightened anxiety and emotional instability, which complicated psychiatric management.

On admission, her vital signs were stable, and there were no significant neurological deficits. Aripiprazole was initiated at a dose of 3 mg/day approximately 24 h after DXM ingestion, to address her severe impulsivity, self‐harming behavior, and depressive symptoms. Within 24 h, the patient developed a fever of 38.6°C, hyperactive tendon reflexes, and hypertonia of the lower extremities. Blood tests showed a significant increase in creatine kinase (CK) levels from 511 IU/L to 7421 IU/L.

The diagnosis for serotonin syndrome was based on the Hunter Serotonin Toxicity Criteria, which require the presence of a serotonergic agent plus specific symptoms, such as spontaneous clonus, inducible clonus with agitation or diaphoresis, ocular clonus, tremor and hyperreflexia, or hypertonia and elevated temperature. In this case, the patient exhibited hyperreflexia, hypertonia, and a temperature of 38.6°C, fulfilling the criteria for serotonin syndrome.

Supportive care, including intravenous hydration and symptomatic management, was initiated, resulting in progressive improvement in the patient's clinical condition. On the 7th day, the tendon reflexes normalized, and on the 14th day, the CK level returned to baseline. At the time of discharge, the neuromuscular symptoms had completely disappeared, and she received extensive psychoeducation on drug misuse and drug safety. With regard to psychological education for addictions, a simple intervention program called “FARPP (First Aid Relapse Prevention Program)” was implemented in the psychiatric emergency ward for drug abuse and dependence. This was a four‐session program.

## DISCUSSION

This case underscores the potential risk of serotonin syndrome when reintroducing antipsychotic medication in a patient with recent DXM misuse. While aripiprazole alone is not a common cause of serotonin syndrome,^8^ its metabolism via CYP2D6 and CYP3A4 suggests a possible interaction with residual DXM metabolites, which may have delayed serotonin clearance and increased the risk of toxicity.

Aripiprazole acts as a partial agonist at serotonin 5‐HT1A receptors and as an antagonist at 5‐HT2A receptors. Overstimulation of 5‐HT1A receptors may have contributed to autonomic symptoms, while muscle rigidity and myoclonus, which depend on 5‐HT2A receptors, were not observed.^9^

The pharmacokinetics of DXM emphasize the importance of allowing a sufficient period of time before reintroducing serotonergic or dopaminergic agents; the plasma half‐life of DXM is approximately 1.5 h, but when combined with drugs that selectively inhibit CYP2D6, the biological half‐life is extended to 29.5 h The biological half‐life is reported to be 29.5 h.^10^

This consideration is particularly critical for individuals with reduced CYP2D6 activity, as the drug's elimination may be substantially delayed in poor metabolizers. For these reasons, it is recommended that patients who have recovered from an overdose of DXM should wait a sufficient amount of time before resuming antipsychotic medication in order to minimize the risk of serotonin toxicity.

## CONCLUSION

This case illustrates the necessity of cautious reintroduction of serotonergic or dopaminergic medications following DXM overdose. Given the potential for prolonged metabolism and interaction—especially in individuals with reduced CYP2D6 activity—clinicians should allow for adequate washout periods before restarting psychotropic agents, such as aripiprazole.

## AUTHOR CONTRIBUTIONS

Naoko Saito contributed to the clinical management of the patient, collected clinical data, and drafted the manuscript. Nobuhiko Noguchi supervised the clinical and academic aspects of the case, critically revised the manuscript, and contributed to the conceptual framework. Maki Toida and Masatoshi Miyauchi were involved in the psychiatric assessment and treatment of the patient and provided critical review of the manuscript. Takeshi Asami contributed to manuscript revision and provided expert guidance on pharmacological mechanisms relevant to the case. All authors read and approved the final manuscript.

## CONFLICT OF INTEREST STATEMENT

The authors declare no conflicts of interest.

## ETHICS APPROVAL STATEMENT

Ethical approval was obtained from the institutional review board, and written informed consent was obtained from the patient's legal guardian.

## PATIENT CONSENT STATEMENT

Written informed consent was obtained from the patient and legal guardian for publication of this case report.

## CLINICAL TRIAL REGISTRATION

N/A.

## Data Availability

Data supporting this study are available upon request from the corresponding author.
